# Activated Ion Electron Capture Dissociation (AI ECD) of Proteins: Synchronization of Infrared and Electron Irradiation with Ion Magnetron Motion

**DOI:** 10.1016/j.jasms.2008.12.015

**Published:** 2009-05

**Authors:** Victor A. Mikhailov, Helen J. Cooper

**Affiliations:** School of Biosciences, College of Life and Environmental Sciences, University of Birmingham, Edgbaston, Birmingham, United Kingdom

## Abstract

Here, we show that to perform activated ion electron capture dissociation (AI-ECD) in a Fourier transform ion cyclotron resonance (FT-ICR) mass spectrometer equipped with a CO_2_ laser, it is necessary to synchronize both infrared irradiation and electron capture dissociation with ion magnetron motion. This requirement is essential for instruments in which the infrared laser is angled off-axis, such as the Thermo Finnigan LTQ FT. Generally, the electron irradiation time required for proteins is much shorter (ms) than that required for peptides (tens of ms), and the modulation of ECD, AI ECD, and infrared multiphoton dissociation (IRMPD) with ion magnetron motion is more pronounced. We have optimized AI ECD for ubiquitin, cytochrome *c*, and myoglobin; however the results can be extended to other proteins. We demonstrate that pre-ECD and post-ECD activation are physically different and display different kinetics. We also demonstrate how, by use of appropriate AI ECD time sequences and normalization, the kinetics of protein gas-phase refolding can be deconvoluted from the diffusion of the ion cloud and measured on the time scale longer than the period of ion magnetron motion.

## Graphical Abstract

Modulation of IR activation and ECD by ion magnetron motion must be taken into account for synchronization of IR-AI ECD of intact proteins.

**Figure:**
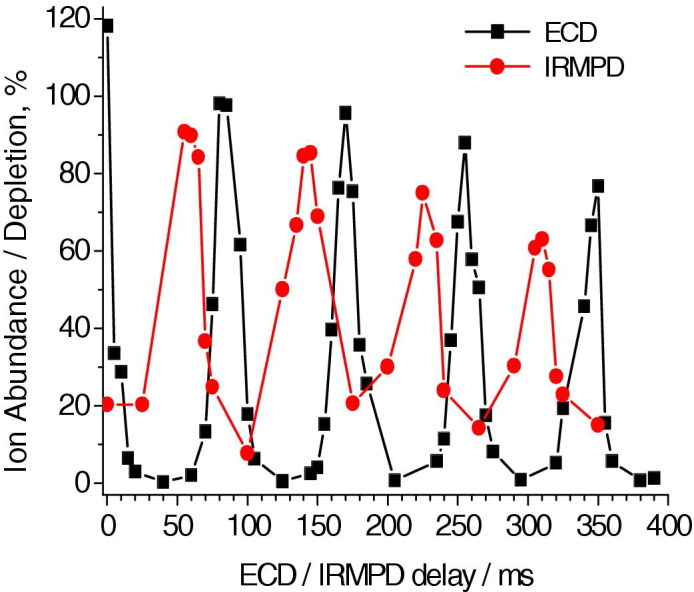

Since its discovery 10 years ago [1], electron capture dissociation (ECD) has become a powerful tool for structural analysis of proteins in both “bottom-up” and “top-down” approaches [2, 3, 4]. Although capture of electrons by cations can be achieved in ion traps [5, 6, 7], ECD is most commonly performed in Fourier transform ion cyclotron resonance (FT-ICR) [8] mass spectrometers. ECD is a fast process, which produces mainly *c′* and *z•* fragment ions via cleavage of N–C_α_ bonds in the protein backbone, and a smaller number of *a•* and *y* fragments [9, 10]. In peptide ECD experiments, radical *c•* and even-electron *z′* fragment ions are also often observed [4, 11, 12, 13]. Those fragments are produced from a short-lived post-ECD complex by H-atom abstraction from the *c′* ion by the α-carbon radical on the *z•* ion. Heating the cloud of trapped parent ions by infrared (IR) radiation before ECD shortens the life time of this complex and decreases the yield of *c•* and *z′* ions in favor of *c′* and *z•* ions [13, 14].

In contrast to threshold tandem mass spectrometry methods such as infrared multiphoton dissociation (IRMPD) [15, 16] and collision-induced dissociation (CID) [17, 18], ECD leaves noncovalent bonds in peptides and proteins intact [19, 20]. Electron capture leads to formation of undissociated charge-reduced ions, where the backbone is cleaved, but the fragments are held together by the noncovalent interactions. These species typically dominate ECD mass spectra. For proteins with masses larger than ca. 20 kDa, ECD has to be facilitated by a slow-heating activation of the protein ions to destroy the noncovalent structure and release the ECD fragments for detection [19, 21, 22]. Such activation is reflected in ECD mass spectra as depletion of the reduced ions and increase in the number of different fragments. It may be achieved by collisions with background gas [19, 22, 23], IR irradiation [20, 22, 23, 24, 25], or increasing the temperature of the ICR cell [20, 23]. These methods, collectively termed activated ion electron capture dissociation (AI ECD) [19], are also required for optimum sequence coverage of smaller integral membrane proteins, which are not produced in high charge states by electrospray ionization (ESI) due to their low hydrophobicity [24]. For some peptides with MW ≤ 5 kDa, AI ECD has also been found to increase fragment ion yield and sequence coverage in comparison with the standard ECD [14, 25, 26].

In addition to improving protein sequence coverage, AI ECD has also been used to study protein tertiary structure. The yield of ECD fragments is expected to be higher from the unfolded parts of the protein with very few or no fragments originating from its tightly folded regions. Fragmentation patterns of different charge states of ubiquitin and cytochrome *c* have been studied under various ECD conditions [1, 4, 20, 27, 28, 29, 30]. McLafferty and coworkers analyzed the yields of *c′, z•* and *a•, y* fragment ions from different parts of ubiquitin cations [27, 28, 29]. The 5+ charge state did not produce any ECD fragments at room temperature, and the 6+ charge state produced ECD fragments only from the regions close to the N- and C-termini. For higher charge states, both ECD yield and the number of cleaved bonds increased with increasing number of protons on the protein. The lower charge states could be partially or completely unfolded by heating the ICR cell up to 175 °C or using IR excitation, with both resulting in a significant increase in ECD efficiency. IR excitation was performed both before and after ECD with similar results. Kinetic measurements on protein refolding after IR activation were performed for the 7+ charge state [27]. The delay between IR activation and ECD was varied, and the ECD yield from different regions of the protein was recorded. ECD experiments on cytochrome *c* revealed a similar relationship between the charge state of the protein and the number of cleaved sites in the backbone [20]. The lower charge states of cytochrome *c* could also be efficiently denatured by thermal or IR activation. The kinetic measurements, however, demonstrated that different charge states of cytochrome *c* were refolding on a much longer (>1 min) timescale than the 7+ ubiquitin ion.

The experiments on ubiquitin and cytochrome *c* described above were carried out using long, 1.2 to 4 s, electron irradiation [20, 27]. Later introduction of indirectly heated dispenser cathodes for electron emission shortened the time required for electron irradiation down to tens of ms and below [25, 31, 32]. Such short times of irradiation are now comparable or smaller than typical periods of ion magnetron motion in the ICR cell. Ion magnetron motion (IMM) is a slow periodic motion around the axis of the ICR trap, whose frequency does not depend on *m/z* [32, 33]. IMM is superimposed on the fast cyclotron motion. As shown by Tsybin et al., optimum ECD conditions can be obtained only by correctly phasing electron injection with IMM [32]. If the delay between ion trapping and electron injection is varied, a modulation of ECD efficiency with IMM periodicity is observed due to the imperfect overlap between the trajectories of the trapped ions and the electron beam. Increasing the number of trapped peptide ions results in a narrower ECD modulation peak width and increases the amplitude of ECD variation within one period [32]. A prerequisite of top-down ECD of intact proteins is the trapping of large numbers of ions. (The number of fragmentation channels is larger than for peptides and the maximum theoretical ECD efficiency is ca. 37% [10]). Modulation of ECD of intact proteins by ion magnetron motion is therefore expected to be pronounced. Furthermore, AI ECD of proteins with IR lasers involves an additional time interval to accommodate the IR activation. In many FT-ICR instruments such as ours (Thermo Finnigan LTQ FT), the IR beam is aligned at an angle to the axis of the ICR cell to avoid collision with the ECD assembly, the latter being usually aligned along the cell axis. Thus not only the electron beam, but also the IR beam may not be perfectly overlapped with the trajectories of the trapped ions.

Here, we investigate the effect of ion magnetron motion on the behavior of ECD, IRMPD, and IR-activated ion ECD (AI ECD) of intact multiply protonated proteins (bovine ubiquitin, cytochrome *c*, and equine apo-myoglobin). We also investigate the effect of ion charge state and intensity of IR activation on the efficiency of fragmentation under AI ECD. Synchronization of IR activation and ECD with IMM also enabled observation of the gas-phase refolding of ubiquitin ions. As the period of ion magnetron motion does not depend on ion *m/z* ratio, our results can be extended to other proteins.

## Experimental

### Materials

Bovine ubiquitin (8.56 kDa, 76 amino acids [AA]) and cytochrome *c* (12.22 kDa, 104 AA), and equine apo-myoglobin (16.95 kDa, 153 AA) were purchased from Sigma Aldrich (Poole, UK) and used without further purification. Methanol (Fisher Scientific, Leicestershire, UK), water (J. T. Baker, Deventer, Netherlands), and formic acid (Fisher Scientific) were used for preparing the electrospray solution. Peptide Substance P (Sigma Aldrich) was used to calibrate the instrument and for comparison with fragmentation of the intact proteins.

### Mass Spectrometry

Protein samples (1–3 μM in 49.5/49.5% of H_2_O/CH_3_OH and 1% of formic acid) were directly infused via an external nanospray ionization source (Advion Biosciences, Ithaca, NY) into a 7T Thermo Finnigan LTQ FT mass spectrometer (Thermo Fisher Scientific, Bremen, Germany). Ion isolation was performed in the front-end linear ion trap. The isolation width was 50 Th. Automatic gain control (AGC) was used to accumulate precursor cations in the ion trap (target 1 × 10^6^, maximum fill time 2 s) before transporting them into the ICR cell with a trapping voltage of 1 V. IR excitation was carried out in the ICR cell using a 75 W in-built CO_2_ laser (Synrad, Mukilteo, WA). IR fluency was controlled via Thermo software and measured as percent of the maximum (75 W). Electrons for ECD were produced by an indirectly heated barium-tungsten cylindrical dispenser cathode (5.1 mm diameter, 154 mm from the cell, 1 mm off axis) (Heat-Wave Labs, Watsonville, CA). The current across the electrode was ∼1.1 A. It is important for the matter of this paper that in our instrument the IR beam crosses the ICR cell at angle in respect to its axis, whilst the electron beam is aligned to be parallel to the axis of the ICR cell. Ten to 250 microscans were averaged for each fragmentation spectrum. Raw MS data were analyzed by use of Xcalibur 2.05 software (Thermo Fisher Scientific), where the Xtract program was used for calculating monoisotopic masses (44% fit factor, 25% remainder). ProSight PTM (https://prosightptm.scs.uiuc.edu) was used to search for *c, z* and *b, y* protein fragment ions. The mass accuracy for the search was set at 10 ppm. The lists of masses from ECD MS/MS spectra were searched both for “standard” *c′*, *z•* ions, and for “hydrogen transfer” *c•*, *z′* fragments. Manual inspection of MS/MS spectra confirmed c•, z′ assignments.

## Results and Discussion

### Effect of Ion Magnetron Motion on IRMPD and ECD

**Figure 1:**
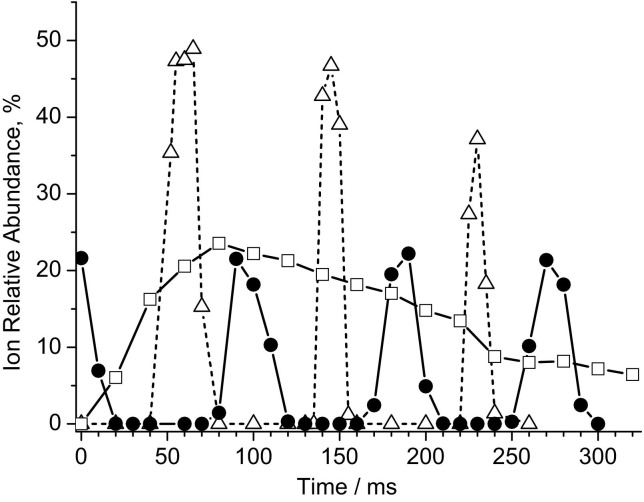
Fragmentation efficiency for the 2+ ion of Substance P with (squares) different durations of ECD; (circles) different ECD time delays with ECD duration 70 ms; and (triangles) different IRMPD delays for IRMPD duration 20 ms.

**Figure 2:**
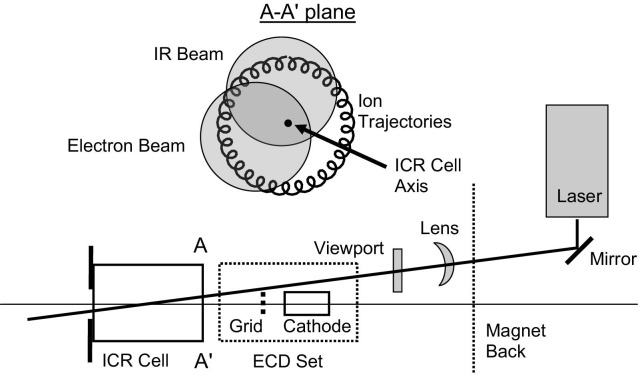
Schematic representation of the geometrical arrangement for electron and IR laser beams in the ICR cell of Thermo-Finnigan LTQ FT. Top: overlap between ion trajectories, electron beam and IR beam in the plane A-A' at the entrance to the ICR cell.

Tsybin et al. observed modulation of ECD by IMM for Substance P and ubiquitin 9+ ions in a homebuilt 9.4 T ESI-Q-FT-ICR mass spectrometer [32]. We have observed similar ECD modulation in our Thermo Finnigan LTQ FT instrument, and, in addition, modulation of IRMPD and infrared-AI ECD efficiencies with the same periodicity. Data for ECD and IRMPD of Substance P are shown in Figure 1. Fragmentation efficiencies were calculated as the ratio of the sum of all fragment ions abundances to the 2+ precursor ion abundance [32]. Time zero in Figure 1 corresponds to a delay of 44.73 ms between ion injection into the ICR cell and the first maximum of ECD efficiency. That time delay was set automatically during program-controlled calibration of ECD and was not changed thereafter. Modulation of ECD efficiency with a period of ca. 85 to 90 ms was observed. The ECD duration was fixed at 70 ms (Figure 1). An irradiation time of 70 ms is typically used for ECD of peptides, because longer irradiation results in secondary, neutralizing electron capture and leads to a decrease in the number of ion fragments and, therefore, ECD efficiency (Figure 1). To synchronize the ECD and IRMPD delays, all our IRMPD measurements for Substance P and the proteins were carried out with both IRMPD and ECD options activated in the controlling software, ECD energy and delay both zeroed, and ECD duration 0.03 ms, the minimum allowed by the software. Our IRMPD data for Substance P and varied IRMPD delay show modulation with the same periodicity as in the ECD data. The width of IRMPD peaks (full width at half maximum peak height) is less than that of ECD peaks because of the shorter duration of IRMPD (20 ms versus 70 ms for ECD). The maxima of IRMPD efficiency are shifted by ca. 30 to 40 ms towards the beginning of the timescale with respect to the maxima of ECD efficiency (Figure 1). The offset between the maxima of the IRMPD and ECD peaks can be explained by the off-axis position of the IR laser beam in the instrument. A schematic representation of the geometrical arrangement of electron and IR laser beams in the ICR cell (55 mm diameter) is given in Figure 2. The diameter of the cathode in the ECD assembly is 10 mm. The diameter of the IR beam is ca. 3.5 mm at the laser entry side, ca. 6 mm at the cell center, and ca. 9 mm at the ion entry side.

As described above, the electron beam is positioned slightly off-axis and aligned parallel to the cell axis. Additionally, the IR beam is deliberately aligned at an angle to the ICR cell axis and crosses the ion trajectories in a place different from their intersection with the electron beam. As the ion cloud moves along the IMM trajectory (counter clockwise in Figure 2, top), first it crosses the IR beam and then the electron beam. Time zero, therefore, corresponds to the first intersection with the electron beam, 44.73 ms after the ions are trapped in the ICR cell. All ECD and IRMPD delays below are given in respect to this time zero.

**Figure 3:**
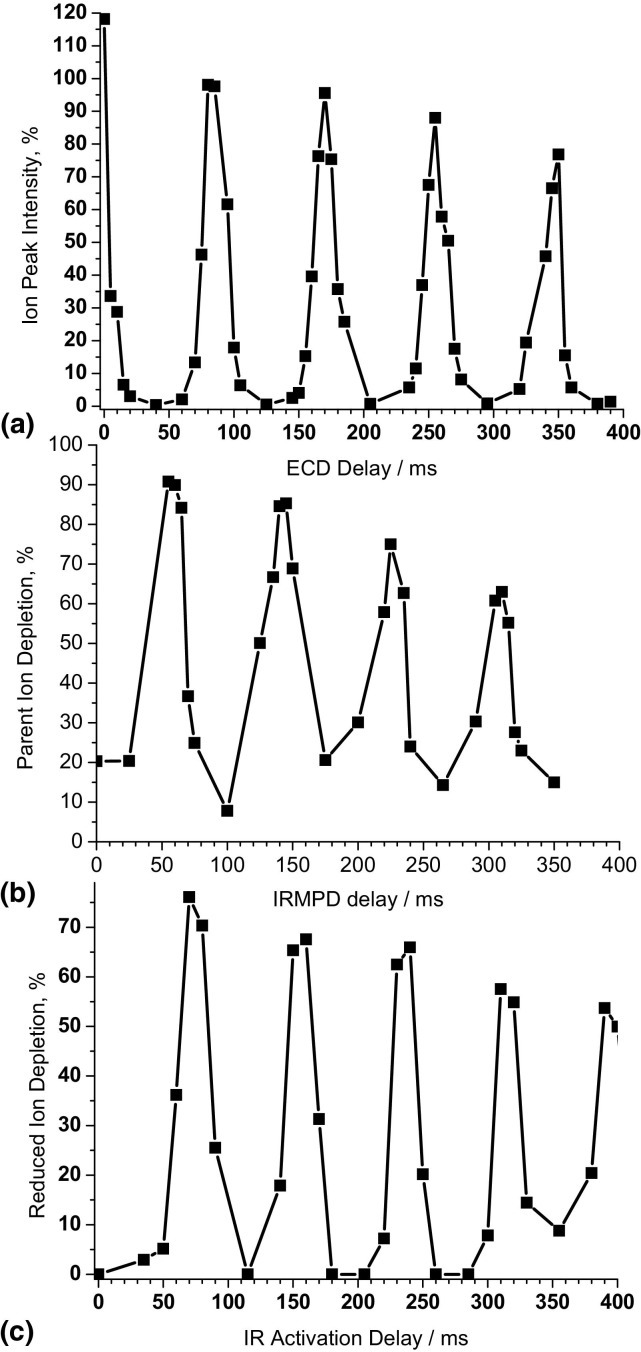
Modulation imposed by ion magnetron motion on (**a**) relative intensities of (M + 6H)^5+•^ reduced ions produced by electron irradiation of ubiquitin (M + 6H)^6+^ ion, (**b**) depletion of the (M + 6H)^6+^ ion abundance by IRMPD, (**c**) post-ECD IR activation: depletion of (M + 6H)^5+•^ reduced ions.

The same modulation of IRMPD and ECD with ca. 85 ms period and 30 ms offset between their maxima was observed for ubiquitin, cytochrome *c* and myoglobin. The results for ECD and IRMPD of 6+ ions of ubiquitin are shown in Figure 3. The relative intensity of the reduced (M + nH)^5+•^ ions was used as a measure of electron capture efficiency (Figure 3a). IRMPD depletion of the precursor ion peak was used as a measure for IRMPD efficiency (Figure 3b). Depletion measurements allow a fast test of the impact of IMM modulation on both ECD and IRMPD, and fast in situ optimization of the time sequence for activated ion ECD of protein ions, as only 10 microscans (transients) are sufficient for each data point. A more appropriate way of calculating absolute ECD and IRMPD efficiencies involves summing the intensities of all fragments, as performed for Substance P (Figure 1). That approach is not suitable for in situ optimization: A much larger number of microscans (transients) would have to be accumulated to provide good signal-to-noise ratio for ECD fragments of intact proteins, and the data analysis would be lengthy (ECD of proteins results in many fragments). The positions of both the ECD and IRMPD maxima coincide for Substance P and ubiquitin, (Figure 1, Figure 3). The positions of the maxima and the modulation period (∼85 ms) also remain the same for cytochrome *c* and myoglobin (data not shown). Ergo, the modulation does not depend on ion *m/z* ratio, as is expected for ion magnetron motion [32, 33]. Due to the geometry of the IR alignment, most of the interaction between the IR beam and the ion population takes place near the middle of the ICR cell (Figure 2). The ions are fast moving in and out of this region because of the z-trapping motion. This motion is at least an order of magnitude faster than IMM [33]. Therefore our results in Figure 1, Figure 3 represent an average over many periods of z-trapping motion.

### Optimization of Activated Ion ECD

The implication of the above findings for implementation of AI ECD on this and similar types of instrument, particularly for protein analysis, is that proper synchronization of ion activation and ECD with the ion magnetron motion must be ensured. It is not possible to carry out ECD and IR activation simultaneously, because the maxima for IR activation do not overlap with those for ECD. IR activation has to be carried out either before or after the ECD event and synchronized with IMM. As mentioned above, effective activation (both pre- and post-ECD) leads to depletion of charge-reduced ions and appearance of new ECD fragments in the MS/MS spectra. Thus depletion of the charge-reduced ions can be used as a measure of activation efficiency. Time-dependent variation of the depletion of reduced (M + 6H)^5+•^ ions under post-ECD activation is presented in Figure 3c. Electrons were injected for 10 ms at time zero for each data point, and the delay for IR irradiation was varied. The data in Figure 3c demonstrate that IMM also modulates the efficiency of AI ECD, and the efficiency maxima for post-ECD activation (Figure 3c) overlap with those for IRMPD (Figure 3b). Alternatively pre-ECD activation can be employed. In this method, IR irradiation (∼70 ms duration) overlaps the first IRMPD efficiency maximum (Figure 3b), and the electron irradiation is carried out after that at the times corresponding to maximum ECD efficiency (Figure 3a).

In general, optimization of activated ion ECD was achieved as follows. First, ECD duration and ECD cathode voltage were tuned to obtain the maximum number of different ECD fragments from the isolated charge state. ECD delay was set at zero, and IR activation was not used in this step. In cases where only a few, or no, fragments could be produced, thus precluding tuning, optimized values for the next highest charge state were used. Typically, the optimized ECD duration was 5 to 10 ms and the ECD cathode voltage was between −2 and −3.5 V. Second, ECD time delay was chosen either for pre-ECD or post-ECD activation. With the exception of the kinetic studies (see below), the time for ECD was set at *t* = 0 (corresponding to the first ECD efficiency maximum) for post-ECD activation; and *t* = 85 ms (corresponding to the second ECD maximum) for pre-ECD activation. Third, the time for IR activation was centered at *t* = 60 ms (corresponding to the first IRMPD efficiency maximum, Figure 2b) for both pre- and post-activation. The duration of IR irradiation was usually in the region 20 to 120 ms, but extension of IR activation over several IRMPD efficiency maxima was also attempted. Fourth, AI ECD MS/MS spectra for different values of IR fluency were acquired. The mass spectra obtained were analyzed and the number of fragments identified by ProSight PTM was plotted versus IR fluency.

### IR-Activated Ion ECD of Ubiquitin

**Figure 4:**
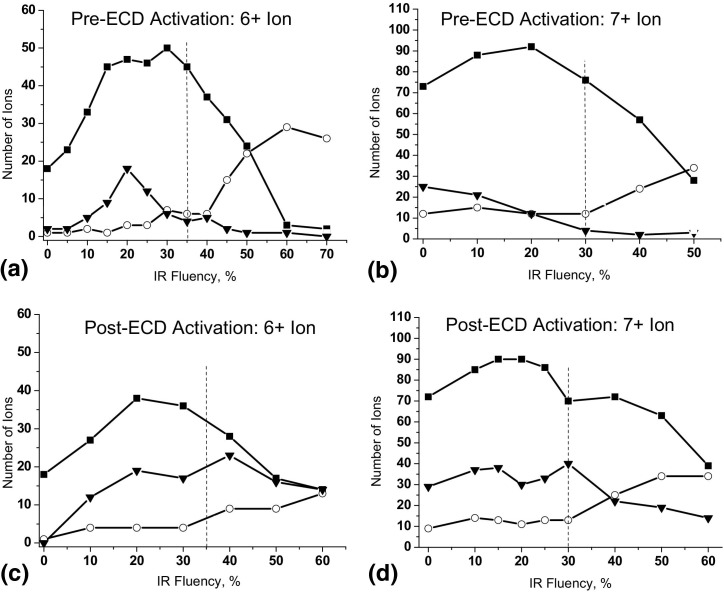
Number of fragments from IR-AI ECD of ubiquitin cations versus laser fluency for (**a**) and (**b**) 85 ms IR activation followed by 10 ms ECD of the 6+ and 7+ ions, respectively, and (**c**) and (**d**) 10 ms ECD followed by 100 ms IR activation of the 6+ and 7+ ions, respectively. Squares represent the number of *c'* and *z•* ions, triangles for *c•* and *z'* ions, and circles for *b* and *y* ions.

Ubiquitin cations in charge states >7+ fragment extensively by ECD without activation. IR activation does not produce new ECD fragments. For charge states ≤ 7+ IR activation leads to the appearance of fragments from new cleavage sites. Figure 4 shows the numbers of different fragment ions from ubiquitin 6+ and 7+ charge states. For pre-ECD activation (Figure 4a and b) the duration of IR irradiation was 85 ms, and ECD duration and delay were 10 ms and 85 ms, respectively. We found that these settings provided a greater (∼15%) total number of fragments than opening a shorter, 30 ms window for IR activation exactly during the first IRMPD efficiency maximum. This improvement could be due to the heating of the ICR cell walls by the IR beam between IRMPD maxima followed by black-body irradiation of the trapped ions from the cell walls. Increased ECD fragmentation in a heated ICR cell was reported by McLafferty and coworkers [20, 27, 28]. For post-ECD activation, ECD duration was 10 ms, and IR irradiation delay and duration were 30 and 100 ms, respectively. Ubiquitin 6+ ions demonstrated poor fragmentation without IR activation: no more than 18 different *c′* and *z•* fragment ions could be produced. Increasing the IR fluency to 30% of the maximum led to an increase in the number of different *c′* and *z•* ions to 50. This was accompanied by a significant increase in the number of *z′* fragments, and a smaller increase in *y* and *c•* fragments. The effect was stronger for pre-ECD activation than for post-ECD activation, with a total of 50 and 41 bonds cleaved, respectively. For the 7+ charge state, IR activation led to ca. 25% increase in the total number of ECD fragments (Figure 4b and d). In all four cases, the optimum IR fluency is 20% to 30% of the maximum. For IR fluency above 30% to 35% of the maximum the number of *c* and *z* fragments diminishes rapidly, and is accompanied by an increase in the number of different *b* and *y* fragments. These threshold values correspond to the onset of IRMPD of (M + 6H)^6+^ and (M + 7H)^7+^ ions.

**Figure 5:**
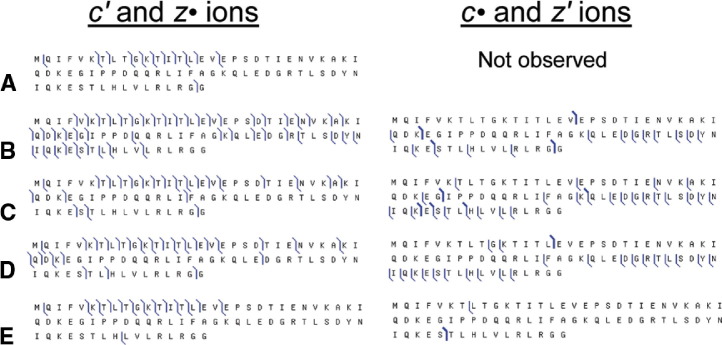
Fragmentation diagrams for (**a**) ECD and (**b**)–(**e**) AI ECD of ubiquitin (M + 6H)^6+^ ion. (**b**) and (**c**) IR activation before ECD, ECD delay 85 ms, IR duration 85 and 25 ms, respectively; (**d**) 100 ms IR activation immediately after ECD; (**e**) IR activation before ECD, ECD delay 1165 ms, IR duration 85 ms.

In all the above cases, *c•* and *z′* ions constitute a significant proportion of the total fragment yield. Figure 5 shows summaries of the fragmentation of ubiquitin 6+ ions for different conditions of IR activation and ECD. Without activation most of the ECD fragments come from the region Val5–Leu15 and the two terminal residues (Figure 5a). IR activation results in fragments from other regions of the protein, (Figure 5b–d). The *z′* ions originate mostly from the region of residues 48–71 for both pre- and post-ECD activation. In the cases of short, 25 ms, pre-ECD activation and long, 100 ms, post-ECD activation, this region of the molecule is underrepresented by “standard” *c′* and *z•* ions, and searching for *z′* ions is essential for achieving maximum sequence coverage (Figure 5c, d).

### IR-Activated Ion ECD of Cytochrome *c* and Myoglobin

Optimization of IR activation was also performed for cytochrome *c* and myoglobin. The results for the 7+, 8+, and 9+ ions of cytochrome *c* and the 11+, 12+, and 13+ ions of myoglobin are presented in Table 1, Table 2. As for ubiquitin, the higher charge states of these two proteins demonstrate extensive bond cleavage under ECD, and IR activation does not increase the number of cleaved bonds. ECD of the lower charge states benefits from IR activation, but the total number of AI ECD fragments decreases with decreasing charge state for z ≤ +9 in the case of cytochrome *c* (Table 1), and z ≤ +12 in the case of myoglobin (Table 2). Optimal IR fluencies range from 20% to 40% depending on the method of activation, protein and its charge state. The threshold to IRMPD of the precursor ions varies between 35% and 40% (Supplemental Figures 1 and 2, which can be found in the electronic version of this article). As has been shown previously, (AI) ECD fragments were not observed from around the region Cys14-Cys17of cytochrome *c* ions, i.e., where the heme group is covalently bound to the protein, (Supplemental Figure 3) [20, 34]. The numbers of *c•* and *z′* fragments produced under IR-AI ECD of cytochrome *c* is larger than that of *c′* and *z•* fragments for all three ions (Table 1). In contrast to ubiquitin, there are more *c•* ions than *z′* ions (Supplemental Figure 3). The reasons for this are not clear. A number of *c•* and *z′* fragments were also produced by AI ECD of myoglobin (Table 2), though their contribution to the total fragment yield is much smaller than in the case of cytochrome *c*. Neither of the two proteins unfolds completely with IR activation, e.g., the Pro100-Gly121 region of the myoglobin 13+ ion remains underrepresented by AI ECD fragments (Supplemental Figure 4). Pre-ECD activation produces a larger overall number of fragments for all three cytochrome *c* charge states, although the increase is marginal for the 9+ charge state. For myoglobin, post-ECD activation produces more fragment ions for the 11+ ion (Table 2).

**Table 1:** Number of fragment ions from IR-AI ECD of cytochrome *c* cations at optimum IR fluency. Numbers of fragment ions at zero IR fluency are given in parentheses

Charge	7+		8+		9+	
Ion type	*c′* and *z^•^*	*c^•^* and *z′*	*c′* and *z^•^*	*c^•^* and *z′*	*c′* and *z^•^*	*c^•^* and *z′*
IR + ECD	12 (0)	22 (1)	19 (1)	41 (6)	45 (34)	57 (41)
ECD + IR	2 (0)	10 (0)	7 (3)	19 (2)	34 (33)	67 (50)

**Table 2:** Number of fragment ions from IR-AI ECD of myoglobin cations at optimum IR fluency. Numbers of fragment ions at zero IR fluency are given in parentheses

Charge	11+		12+		13+	
Ion type	*c′* and *z^•^*	*c^•^* and *z'*	*c′* and *z^•^*	*c^•^* and *z'*	*c′* and *z^•^*	*c^•^* and *z'*
IR + ECD	29 (5)	13 (0)	65 (31)	15 (12)	74 (48)	13 (13)
ECD + IR	28 (4)	21 (5)	51 (34)	20 (18)	43 (36)	18 (12)

### General Features in IR-AI ECD

The decrease in ECD efficiency of protein ions with decreasing charge state has been well documented in the literature [20, 27, 28, 29, 30, 35], and is explained by a decrease in Coulomb repulsion energy available for repelling complementary protein fragments and simultaneous increase in the number of noncovalent bonds holding the fragments together. Our data indicate that there are also limitations on protein unfolding by IR activation, which depend on the *m/z* value of the protein ion. It was not possible to cleave more than 10% of the backbone bonds for charge states below 5+ for ubiquitin, 7+ for cytochrome *c*, and 10+ for myoglobin, i.e., for charge states with less than one proton per ∼15 amino acids. For those charge states for which ECD fragmentation benefits from IR activation, the optimum IR fluency is 20% to 40% of the maximum, i.e., 50% to 100% of the threshold to IRMPD of the precursor ions. In most cases, pre-ECD activation resulted in higher efficiency in terms of the number of N–C_α_ bonds cleaved. Increasing the duration of IR activation to overlap several IRMPD efficiency maxima did not lead to more efficient AI ECD fragmentation. The only result of such increase was lowering the IR fluency at which IRMPD of the precursor ion started. That result is expected for a threshold fragmentation technique, such as IRMPD, where the threshold to fragmentation can be reached either in “high-heat” regime over a short period of time, or in “low-heat” regime over a longer period.

The results show that the ratio of the number of *c•* and *z′* fragments to that of *c′* and *z•* fragments is larger for post-ECD activation than for pre-ECD activation. That observation can be explained by the different fragmentation mechanisms of these two methods. In pre-ECD dissociation, the even-electron precursor ion is first unfolded by IR activation, and then subjected to ECD. ECD fragments are released almost immediately after electron capture, and there exists very little time for H• transfer between *c′* and *z•* fragments. In post-ECD activation, charge-reduced noncovalent complexes of ECD fragments are formed, and after some delay, dissociated by IR activation. Survival of the radical complex facilitates H• transfer and the production of *c•* and *z′* ions.

### Diffusion of the Ion Cloud and Protein Refolding in the ICR Cell

**Figure 6:**
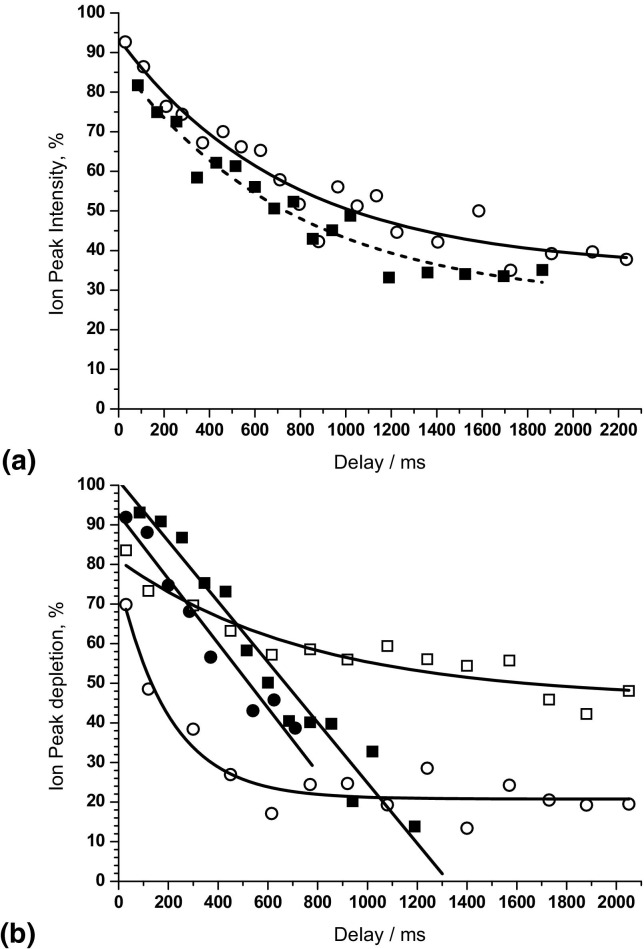
Long-time evaluation of the efficiencies of electron capture, IRMPD and IR-AI ECD for ubiquitin ions. (**a**) (Squares) intensity of (M + 6H)^5+•^ reduced ion produced by electron capture from (M + 6H)^6+^ ion versus ECD delay; (**a**) (circles) IRMPD depletion of ubiquitin (M + 6H)^6+^ ion versus IRMPD delay; (**b**) (filled squares) pre-ECD IR activation: depletion of (M + 6H)^5+•^ reduced ion versus ECD delay; (**b**) (filled circles) pre-ECD IR activation: depletion of (M + 6H)^4+••^ reduced ion versus ECD delay; (**b**) (hollow squares) post-ECD IR activation: depletion of (M + 6H)^5+•^ reduced ion versus IR activation delay; (**b**) (hollow circles) post-ECD IR activation: depletion of (M + 6H)^4+••^ reduced ion versus IR activation delay.

Both electron capture and IRMPD efficiencies decrease with time (Figure 3). ECD and IRMPD efficiencies over a longer time scale are presented in Figure 6a. The time intervals for ECD or IRMPD were set at each of the subsequent ECD or IRMPD maxima. Durations were constant for all measurement points (10 ms for ECD and 25 ms for IRMPD). Both the electron capture and IRMPD efficiencies decrease with time at approximately the same rate. Exponential fits for the curves in Figure 5a give decay times of 765 ± 165 ms for the ECD data and 740 ± 140 ms for the IRMPD data. These values are very similar indicating that the effect causing the decrease in efficiencies is the same for ECD and IRMPD. That effect is most probably diffusion of the ion cloud in the ICR cell caused by Coulombic repulsion between the trapped cations. As the ion cloud spreads in space and time, fewer ions are subjected to electron or IR irradiation.

As described above, depletion of the charge-reduced ions in ECD mass spectra can be used as a measure of overall protein unfolding following IR activation. In pre-ECD activation, if the time delay between IR activation and ECD is increased, more time exists for the protein to refold before electron capture. The newly (re)formed noncovalent bonds should prevent ECD fragments from separating, leading to an increase in intensity of the charge-reduced ions. Thus measuring depletion of the reduced ions for different delays between the IR activation and the ECD event can be used for monitoring protein refolding in the gas-phase. To account for the decrease in electron capture efficiency caused by ion cloud diffusion, the intensities of charge-reduced ions with and without IR activation must be measured at the same ECD delay.

Using slow ECD (1.2 s duration), McLafferty and coworkers demonstrated that the 7+ ions of ubiquitin refold after IR excitation in 1 to 2 s [26]. However, in our instrument, these ions fragment extensively in 10 ms ECD indicating that they are unfolded. We, therefore, used ubiquitin (M + 6H)^6+^ ions in our kinetic measurements. IR activation was centered at the first IRMPD efficiency maximum (Figure 3b) with IR fluency at 30%. The time points for ECD were set at each of the subsequent ECD efficiency maxima (Figure 3a). The results of these measurements are shown in Figure 6b. The depletion of the (M+6H)^5+•^ reduced ion drops from 93 to 13% over a period of 1.2 s, which is close to the refolding time of the 7+ ion found by McLafferty and coworkers [27]. The depletion of the (M + 6H)^4+••^ reduced ion decreases with ECD delay at the same rate. The comparable behavior of the (M + 6H)^5+•^ and (M + 6H)^4+••^ ions is because their depletion in ECD spectra is affected by refolding of the same (M + 6H)^6+^ precursor ions. For ECD delays greater than 1 s, AI ECD fragments are formed mostly from the same segments of the molecule as for ECD without activation, (Figure 5e). That observation suggests that the ubiquitin 6+ ion folds back to the same, or similar, conformation as it had before the activation.

As discussed above, post-ECD activation probes the structure of the backbone-cleaved radical complex held together by noncovalent interactions. The efficiency by which the fragments are released from the complex is affected by the time delay between ECD and IR activation only if new covalent bonds form between the backbone fragments within the charge- reduced ion. Formation of such bonds in the complex should be possible because the complex contains radical *z•* or *c•* ions. New covalent bonds cannot be destroyed by IR activation when it is carried out below the threshold to IRMPD. Data for depletion of (M + 6H)^5+•^ and (M + 6H)^4+••^ ions by post-ECD IR activation for different ECD-IR delays are shown in Figure 6b. ECD duration was 10 ms. IR activation duration was 70 ms and coincided with the subsequent IRMPD efficiency maxima (Figure 3b). IR fluency was 30%. The depletion for each ECD delay was measured against the intensities of charge-reduced ions without activation for zero ECD delay, i.e., the effect of ion diffusion should be reflected in the measurements. The depletion of charge-reduced ions (Figure 6b) does decrease with increasing ECD-IR delay. However, the rate of depletion of (M+6H)^5+•^ ions is less than that observed in the IRMPD of (M+6H)^6+^ ions, (Figure 6a). The difference in the diffusion rate can be explained by a smaller charge on the (M+6H)^5+•^ ions c.f. the (M + 6H)^6+^ ions. The force of Coulomb repulsion between ions is proportional to the square of the charge of the ion, therefore ions in the higher 6+ charge state experience stronger repulsion and their diffusion should happen faster than that of the ions with 5+ charges. Low rate of decrease in the depletion of (M + 6H)^5+•^ ions with the IR activation delay indicates that there is no new chemical bond formation in these radical ions. In the case of doubly-charge-reduced (M + 6H)^4+••^ ions, the rate of depletion is much greater than that for both (M + 6H)^6+^ and (M + 6H)^5+•^ ions, i.e., occurs faster than diffusion. The increasing stability of the (M + 6H)^4+••^ ion may be an indication of the formation of new covalent bonds within this biradical cationic complex between the ECD event and IR activation. Our results are in accordance with those of Kleinnijenhuis et al., who reported similar recombination of two unpaired electrons and formation of new chemical bonds in cationic biradicals of lacticin 481 following double electron capture [36].

## Conclusions

In our study for Substance P, ubiquitin, cytochrome *c*, and myoglobin the period of ion magnetron motion was ca. 85 ms for 1 V trapping voltage in the ICR cell of a Thermo Finnigan LTQ FT mass spectrometer. Periodic modulation of IR activation and ECD by ion magnetron motion should be the same for other proteins because of its invariance in respect to ion *m/z* value. Either pre-ECD or post-ECD IR activation can be employed for those protein ions, which require additional vibrational excitation to destroy their internal noncovalent bonds. These two methods of activation are physically different in respect to the ions they affect, and demonstrate different kinetics. However both methods require precise synchronization of both ECD event and IR activation with the ion magnetron motion. The optimum fluency for IR activation depends on the nature of the protein and its charge state. In our study it ranges from ca. 50% to 100% of the value for the threshold of IRMPD of the precursor (M + nH)^n+^ ion. In the search for fragments, *c•* and *z′* ions should not be overlooked, as they may contribute significantly to the total number of fragments and also bear information from those segments of the protein, which are underrepresented by the other types of ion fragments. Kinetic experiments on proteins in the ICR cell on a time scale larger than IMM period should be performed only after careful selection of the time windows in respect to the ion magnetron motion. Kinetic information must also be deconvoluted from the diffusion of ion cloud in the cell.

## Supplementary data

Supplemental Figures 1 through 4
