# Supplementary material for: Activated Ion Electron Capture Dissociation (AI ECD) of Proteins: Synchronization of Infrared and Electron Irradiation with Ion Magnetron Motion
Source: J Am Soc Mass Spectrom. 2009 May;20(5):763–71. doi: 10.1016/j.jasms.2008.12.015 (PMC2674148; doi:10.1016/j.jasms.2008.12.015)

**Supplemental Figure Legends**

**Supplemental Figure 1.** Fragment numbers from IR-AI ECD of cytochrome c cations *versus* laser fluency for (**a**), (**b**) and (**c**) 85 ms IR activation followed by 6 ms ECD of the 7+, 8+ and 9+ ions, respectively, and (**d**), (**e**) and (**f**) 6 ms ECD of the 7+, 8+ and 9+ ions, respectively, followed by 100 ms IR activation. Squares represent the number of *c'* and *z•* ions, triangles for *c•* and *z'* ions, and circles for *b* and *y* ions.

**Supplemental Figure 2.** Fragment numbers from IR-AI ECD of myoglobin cations *versus* laser fluency for (**a**), (**b**) and (**c**) 85 ms IR activation followed by 5 ms ECD of the 11+, 12+ and 13+ ions, respectively, and (**d**), (**e**) and (**f**) 5 ms ECD of the 11+, 12+ and 13+ ions, respectively, followed by 100 ms IR activation. Squares represent the number of *c'* and *z•* ions, triangles for *c•* and *z'* ions, and circles for *b* and *y* ions.

**Supplemental Figure 3.** Fragmentation diagram for cytochrome c 9+ ion: 85 ms IR activation followed by 10 ms ECD. Highlighted amino acid residues are related to protein acetylation at N-terminus (Gly1), and covalent binding of the heme group (Cys14 and Cys17).

**Supplemental Figure 4.** Fragmentation diagram for myoglobin 13+ ion: 85 ms IR activation followed by 10 ms ECD.

Supplemental Figure 1

Supplemental Figure 2

Supplemental Figure 3

*c'* and *z•* ions

*c•* ions

*z'* ions


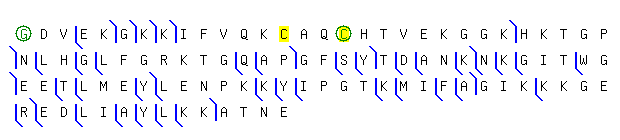

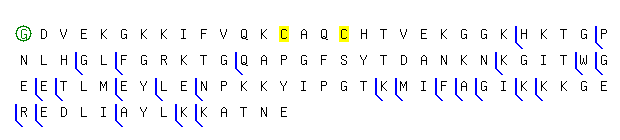

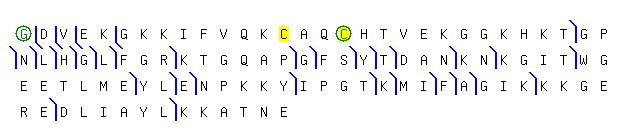


Supplemental Figure 4

*c'* and *z•* ions

*c•* ions

*z'* ions


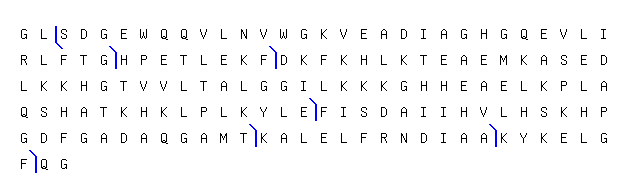

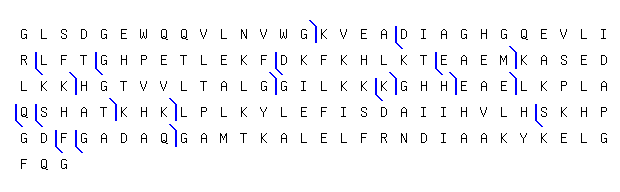

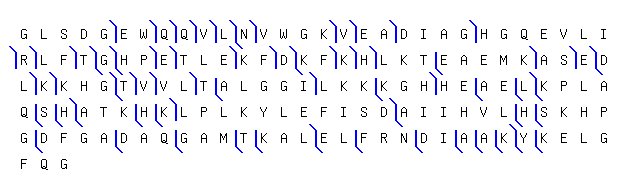

Supplement: Supplemental Figures 1 through 4 [file mmc1.doc]
